# Phenotypic Variability in Vitamin D–Dependent Rickets Type 1a: A Case Report of Two Children With the Same CYP27B1 Mutation

**DOI:** 10.1155/crpe/5823067

**Published:** 2026-07-31

**Authors:** Ali Saleh Alquraishi

**Affiliations:** ^1^ Armed Forces Hospital Southern Region (AFHSR), Khamis Mushait, Saudi Arabia

## Abstract

**Background:**

Vitamin D–dependent rickets Type 1A (VDDR1A) is a rare autosomal recessive disorder caused by pathogenic variants in CYP27B1, resulting in impaired renal 1α‐hydroxylation of 25‐hydroxyvitamin D. The consequent deficiency of active vitamin D disrupts calcium–phosphate homeostasis and causes rickets. Although VDDR1A is monogenic, clinical severity may vary considerably even among patients with the same mutation.

**Case Presentation:**

We describe two unrelated Saudi boys with genetically confirmed VDDR1A who both harbored the same homozygous CYP27B1 c.1286G > C (p.Arg429Pro) variant but exhibited markedly different clinical courses. The first child presented at 7 months of age with hypocalcemic seizures and classical radiographic features of rickets but preserved growth. Early initiation of alphacalcidol and calcium supplementation was associated with biochemical improvement, resolution of seizures, and favorable clinical recovery by 2.5 years of age. In contrast, the second child was diagnosed at 16 months after developmental delay, recurrent respiratory infections, growth failure, and early skeletal deformities. Long‐term follow‐up into adolescence revealed severe bowing deformities, kyphoscoliosis, osteopenia, and healed fractures in the setting of delayed diagnosis and inconsistent treatment adherence, despite receiving the same therapy.

**Conclusion:**

These cases highlight substantial phenotypic variability in VDDR1A even in children harboring the same CYP27B1 mutation. Early recognition and sustained treatment with active vitamin D therapy are crucial to prevent severe skeletal complications and optimize growth, particularly in populations with high rates of consanguinity.

## 1. Introduction

Vitamin D is essential for calcium–phosphate homeostasis, endochondral ossification, and normal skeletal mineralization. It is acquired through dietary intake or synthesized in the skin after ultraviolet exposure, but in either form it requires sequential activation by cytochrome P450 enzymes [1]. The first hydroxylation occurs in the liver, generating 25‐hydroxyvitamin D [25(OH)D], followed by renal 1α‐hydroxylation by the mitochondrial enzyme 25‐hydroxyvitamin D‐1α‐hydroxylase, encoded by CYP27B1, to produce 1,25‐dihydroxyvitamin D [1,25(OH)_2_D], the biologically active hormone [2, 3]. Through its actions on intestinal calcium and phosphate absorption and skeletal mineralization, 1,25(OH)_2_D plays a central role in maintaining normal growth‐plate architecture and bone health [4].

Disruption of this pathway results in impaired mineral homeostasis and defective mineralization of growing bone, causing rickets. Although nutritional vitamin D deficiency remains the most common cause worldwide, inherited defects in vitamin D metabolism or signaling account for a small but clinically important subset of cases. Vitamin D–dependent rickets Type 1A (VDDR1A) is an autosomal recessive disorder caused by biallelic pathogenic variants in CYP27B1, leading to deficient or absent 1α‐hydroxylase activity [5, 6]. As a consequence, affected children typically have low or undetectable serum 1,25(OH)_2_D despite normal or near‐normal 25(OH)D concentrations, together with hypocalcemia, hypophosphatemia, secondary hyperparathyroidism, elevated alkaline phosphatase, and radiographic features of rickets [7]. Clinically, VDDR1A usually presents in infancy or early childhood with hypotonia, delayed motor development, growth failure, skeletal deformities, and, in some cases, hypocalcemic seizures [7].

More than 50 CYP27B1 variants have been described across diverse populations, and phenotypic expression can vary widely even when the underlying genotype is identical [8, 9]. In the Saudi population, a recent molecular series identified c.1286G > C (p.Arg429Pro) as the most frequent CYP27B1 variant, occurring in 22 patients from 15 unrelated families, suggesting a possible founder effect [9]. Although genotype–phenotype variability in VDDR1A has been recognized, the factors underlying this variability remain incompletely understood. In addition to the underlying molecular defect, age at diagnosis, severity of biochemical derangement at presentation, treatment adherence, calcium intake, comorbid conditions, and possibly genetic or environmental modifiers may all influence disease expression and outcome [10, 11]. Importantly, early treatment with active vitamin D therapy, typically calcitriol or alphacalcidol, together with calcium supplementation can correct biochemical abnormalities and promote skeletal healing, whereas delayed diagnosis or poor adherence may result in persistent deformities, fractures, impaired growth, and long‐term disability [12].

Here, we report two unrelated Saudi boys with VDDR1A caused by the same homozygous CYP27B1 variant, c.1286G > C (p.Arg429Pro), who exhibited markedly divergent clinical severity and radiographic outcomes. By contrasting an infant diagnosed early and treated consistently with another child diagnosed later and managed with poor long‐term adherence, this report highlights how the clinical expression of VDDR1A may differ substantially despite an identical genotype. It also underscores the importance of early recognition, sustained treatment, and close long‐term follow‐up, particularly in populations with a high prevalence of consanguinity.

## 2. Case Description

**FIGURE 1:**
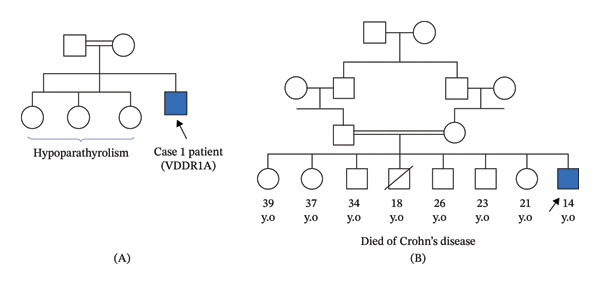
Family pedigrees of two unrelated patients with vitamin D–dependent rickets Type 1A (VDDR1A). Note: (A) The pedigree of Case 1. The proband is indicated by a blue shaded square. The parents are consanguineous, represented by a double horizontal line. Three female siblings are shown as unaffected. The label “hypoparathyroidism” reflects a family diagnosis reported in a relative but is not represented by a specific pedigree symbol. (B) The pedigree of Case 2. The proband is similarly indicated by a blue shaded square.

**FIGURE 2:**
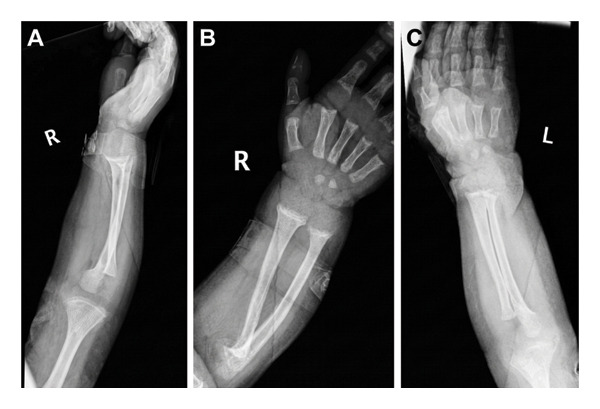
Baseline wrist and forearm radiographs of Case 1 demonstrating rachitic changes at presentation. Note: (A–C) Radiographs obtained at 7 months of age demonstrating widening of the distal radial and ulnar growth plates with metaphyseal cupping and fraying, consistent with active rickets. Panel (A) shows a lateral view of the right forearm and wrist, panel (B) shows an anteroposterior view of the right wrist/hand, and panel (C) shows a left wrist/forearm view.

**FIGURE 3:**
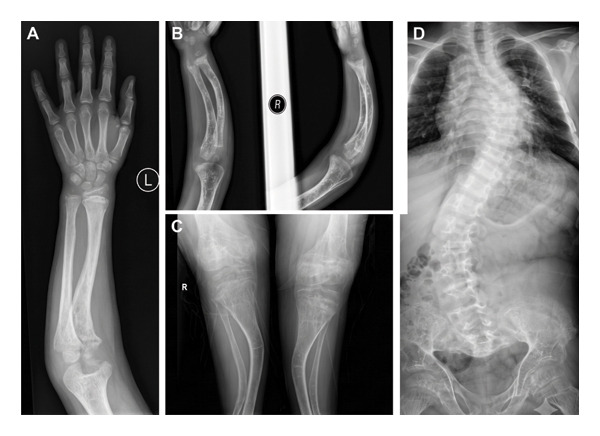
Serial radiographic manifestations of severe progressive vitamin D–dependent rickets Type 1A (VDDR1A) in Case 2. Note: (A–D) Representative serial radiographs illustrating the multisite skeletal manifestations of severe VDDR1A over time. Panel (A) (age 6 years) shows persistent rachitic changes at the wrist, including metaphyseal irregularity and osteopenia. Panel (B) (age 33 months) demonstrates marked bilateral bowing deformity of the forearms with diffuse osteopenia. Panel (C) (age 12 years) shows bilateral lower‐limb bowing deformities with persistent skeletal abnormalities. Panel (D) (age 12 years) demonstrates severe axial skeletal involvement with thoracolumbar scoliosis.

Two unrelated Saudi boys were evaluated for VDDR1A and were found to harbor the same homozygous pathogenic CYP27B1 variant, c.1286G > C (p.Arg429Pro). Despite the identical molecular diagnosis, the two patients exhibited markedly different clinical severity, skeletal burden, and treatment outcomes. Family pedigrees are shown in Figure 1, while the principal clinical, biochemical, and radiographic findings are summarized in Table 1. Baseline radiographs of Case 1 are presented in Figure 2, and serial radiographs of Case 2 are shown in Figure 3.

**TABLE 1:** Comparative clinical, biochemical, and radiologic characteristics of two unrelated patients with vitamin D–dependent rickets type 1A (VDDR1A).

Parameter	Case 1 (boy, latest follow‐up at 2.5 y)	Case 2 (boy, latest follow‐up at 14 y)
Age at diagnosis	7 months	16 months
Presenting symptoms	Two brief tonic convulsions	Delayed dentition, delayed sitting/walking, recurrent chest infections, failure to thrive
Growth status at evaluation	Height and weight approximately at the 50th percentile	Height and weight < 1st percentile
Key clinical signs	Frontal bossing, widened anterior fontanel, hypotonia, wrist widening, protuberant abdomen	Severe lower‐limb bowing, kyphoscoliosis, frontal bossing, Harrison groove, rachitic rosary, muscle weakness, protuberant abdomen
Radiologic findings before treatment	Widening of the distal radial and ulnar physes with metaphyseal cupping and fraying, consistent with active rickets	Diffuse osteopenia, metaphyseal widening/irregularity, bilateral forearm deformity, lower‐limb bowing, and chronic skeletal changes consistent with longstanding rickets
Radiologic findings on follow‐up	Improvement of metaphyseal cupping and fraying with radiographic healing after treatment	Persistent osteopenia/osteoporosis, severe bowing deformities of the forearms and lower limbs, and kyphoscoliotic deformity despite prolonged treatment
Calcium (mmol/L)	1.56 ⟶ 1.9	1.67 ⟶ 2.2
Phosphate (mmol/L)	1.13 ⟶ 1.1	0.77 ⟶ 1.23
ALP (U/L)	747 ⟶ 523	1769 ⟶ 442
PTH (pmol/L)	78 ⟶ 59	158 ⟶ 42
25‐OH vitamin D (ng/mL)	84.6	27.3 ⟶ 22.8
1,25‐(OH)_2_ vitamin D (pg/mL)	35 (low)	Undetectable
Renal ultrasound	Unremarkable	Both kidneys unremarkable; right 72 × 30 mm, left 76 × 44 mm
Genetic variant	Homozygous *CYP27B1* c.1286G > C (p.Arg429Pro)	Homozygous *CYP27B1* c.1286G > C (p.Arg429Pro)
Treatment	Alphacalcidol plus elemental calcium	Alphacalcidol plus elemental calcium (poor long‐term adherence)
Response to therapy	Marked biochemical, clinical, and radiographic improvement	Partial biochemical improvement, but persistent severe skeletal deformities due to delayed diagnosis and poor adherence

*Note:* Values are presented as baseline ⟶ follow‐up where serial measurements were available. ALP, alkaline phosphatase; PTH, parathyroid hormone.

### 2.1. Case 1

Case 1 was a Saudi boy who first presented at 7 months of age after experiencing two brief tonic convulsions, prompting investigation for an underlying metabolic disorder. Clinical examination revealed several classical manifestations of rickets, including frontal bossing, a widened anterior fontanel, hypotonia, wrist widening, and a protuberant abdomen. Despite these skeletal features, his linear growth and weight gain were preserved, both tracking at approximately the 50th percentile for age, indicating that significant growth failure had not yet developed at the time of diagnosis.

Initial biochemical evaluation showed hypocalcemia (serum calcium 1.56 mmol/L), hypophosphatemia (serum phosphate 1.13 mmol/L), markedly elevated alkaline phosphatase (747 U/L), and secondary hyperparathyroidism (PTH 78 pmol/L). Serum 25‐hydroxyvitamin D was within the normal range (84.6 ng/mL), whereas serum 1,25‐dihydroxyvitamin D was low (35 pg/mL), a biochemical profile strongly suggestive of impaired renal 1α‐hydroxylation rather than nutritional vitamin D deficiency. Renal ultrasonography (KUB) was unremarkable.

Baseline radiographs obtained at 7 months of age demonstrated active rachitic changes involving both wrists and forearms (Figure 2). These included widening of the distal radial and ulnar growth plates with metaphyseal cupping and fraying, without documented fracture. Figure 2A shows a lateral view of the right forearm and wrist, Figure 2B shows an anteroposterior view of the right wrist/hand, and Figure 2C shows a left wrist/forearm view.

Molecular testing confirmed a homozygous CYP27B1 c.1286G > C (p.Arg429Pro) pathogenic variant, establishing the diagnosis of VDDR1A. The patient was started promptly on alphacalcidol together with elemental calcium supplementation and remained adherent to treatment. During follow‐up, his biochemical parameters improved, with serum calcium increasing to 1.9 mmol/L and alkaline phosphatase decreasing to 523 U/L, accompanied by clinical improvement and no further convulsions. By 2.5 years of age, his skeletal manifestations had improved clinically, illustrating the favorable effect of early diagnosis and sustained treatment in VDDR1A.

### 2.2. Case 2

Case 2 was a Saudi boy with a substantially more severe and protracted clinical course. He was diagnosed at 16 months of age after progressive skeletal and developmental abnormalities became evident. His early manifestations included delayed dentition, delayed sitting and walking, recurrent chest infections, and failure to thrive. Over time, his course was further complicated by frequent bronchial asthma exacerbations and fractures after minor trauma, suggesting longstanding bone fragility. Family history revealed parental consanguinity and the death of one sibling from Crohn’s disease, although no other relatives were known to have rickets (Figure 1).

At 14 years of age, he had severe chronic skeletal deformities, including marked bilateral lower‐limb bowing, kyphoscoliosis, frontal bossing, Harrison groove, rachitic rosary, generalized muscle weakness, and a protuberant abdomen. His height and weight were both below the 1st percentile, consistent with profound long‐term growth impairment.

Biochemical evaluation was consistent with severe VDDR1A and showed more pronounced abnormalities than in Case 1. At diagnosis, serum calcium was 1.67 mmol/L, phosphate 0.77 mmol/L, alkaline phosphatase 1769 U/L, and PTH 158 pmol/L. Serum 25‐hydroxyvitamin D was mildly reduced (27.3 ng/mL, later 22.8 ng/mL), whereas 1,25‐dihydroxyvitamin D was undetectable, supporting defective 1α‐hydroxylase activity. Renal ultrasonography showed no structural renal abnormality; the right kidney measured 72 × 30 mm and the left 76 × 44 mm, while the urinary bladder was inadequately filled at the time of examination.

Serial radiographs documented extensive and progressive multisite skeletal involvement (Figure 3). Panel A, obtained at 6 years of age, demonstrates persistent wrist rachitic changes with metaphyseal irregularity and osteopenia. Panel B, obtained at 33 months, shows marked bilateral bowing deformity of the forearms with diffuse osteopenia. Panel C, obtained at 12 years, demonstrates bilateral lower‐limb bowing deformities with persistent skeletal abnormalities. Panel D, also obtained at 12 years, shows severe axial skeletal involvement with thoracolumbar scoliosis. Collectively, these radiographs reflect the chronic skeletal burden of longstanding, poorly controlled disease.

Genetic analysis confirmed the same homozygous CYP27B1 c.1286G > C (p.Arg429Pro) pathogenic variant identified in Case 1. He was treated with alphacalcidol and elemental calcium supplementation; however, long‐term poor adherence substantially limited therapeutic benefit. Although some biochemical improvement was observed during follow‐up (calcium 2.2 mmol/L, phosphate 1.23 mmol/L, alkaline phosphatase 442 U/L, and PTH 42 pmol/L), the delayed diagnosis and inconsistent treatment were associated with persistent deformities, severe growth failure, and irreversible skeletal sequelae.

## 3. Discussion

VDDR1A is an autosomal recessive disorder caused by pathogenic variants in CYP27B1, which encodes renal 25‐hydroxyvitamin D‐1α‐hydroxylase, the enzyme responsible for conversion of 25‐hydroxyvitamin D to the active hormone 1,25‐dihydroxyvitamin D [12]. Deficiency of this enzyme impairs intestinal calcium and phosphate absorption and disrupts endochondral mineralization, resulting in hypocalcemia, hypophosphatemia, secondary hyperparathyroidism, elevated alkaline phosphatase, and radiographic rickets [12]. Both children in the present report exhibited the characteristic biochemical and radiographic profile of VDDR1A, and the diagnosis was confirmed molecularly by identification of the same homozygous CYP27B1 variant, c.1286G > C (p.Arg429Pro).

The CYP27B1 c.1286G > C (p.Arg429Pro) variant identified in both of our patients has been previously reported in Saudi patients with VDDR1A. Importantly, a recent Saudi molecular cohort identified this variant as the most common CYP27B1 mutation, present in 22 patients from 15 unrelated families, supporting a possible founder effect in this population [8]. This observation places our cases within an established regional genetic background and supports the clinical relevance of recognizing VDDR1A in consanguineous populations. Although genotype–phenotype variability in VDDR1A is increasingly recognized, reports describing markedly different outcomes in patients with the same CYP27B1 variant remain limited. Our two cases highlight that the same homozygous pathogenic variant can lead to very different skeletal severity, growth outcomes, and long‐term prognosis. In Case 1, early diagnosis and consistent treatment with alphacalcidol and calcium led to biochemical improvement, seizure resolution, and a favorable outcome. In contrast, Case 2 had delayed diagnosis and a more severe chronic course, with longstanding skeletal disease, poor growth, and persistent deformities despite treatment.

The contrasting courses of the two boys suggest that timing of diagnosis and treatment adherence were key determinants of outcome. In VDDR1A, early diagnosis and prompt active vitamin D therapy can restore calcium–phosphate balance, improve growth‐plate mineralization, and prevent severe skeletal deformities [10, 11]. By contrast, prolonged untreated disease during early childhood can lead to progressive bowing, fractures, vertebral deformity, and impaired growth. This likely explains the severe phenotype in Case 2, who had delayed diagnosis, developmental delay, recurrent chest problems, marked limb deformities, and later kyphoscoliosis, with poor long‐term adherence further limiting recovery. Severe presentations associated with the same p.Arg429Pro variant have been reported previously, including generalized hypotonia with respiratory failure requiring intensive care support, indicating that this genotype may be associated with a broad phenotypic spectrum rather than a uniform clinical course [9].

At the same time, the phenotypic contrast between the two cases should not be attributed solely to delayed diagnosis and poor adherence. Clinical expression in VDDR1A is likely multifactorial. Previous studies have shown considerable heterogeneity in age at presentation, biochemical severity, growth failure, fracture burden, and long‐term skeletal sequelae even among patients with the same disorder [10, 11, 13]. In addition to treatment timing and continuity, several other factors may contribute to this variability, including differences in residual enzyme activity associated with specific alleles, modifier genes affecting vitamin D signaling or calcium–phosphate regulation, nutritional calcium intake, baseline vitamin D status, sun exposure, and comorbid illnesses that may influence growth, mobility, or adherence to therapy [13, 14]. In Case 2, recurrent respiratory morbidity, fractures, and chronic musculoskeletal disability may themselves have compounded the clinical course by reducing mobility, impairing nutrition, and making long‐term disease control more difficult. Thus, our cases support the concept that phenotype in VDDR1A reflects the interaction between genotype and modifiable clinical factors rather than genotype alone [14].

These observations are also clinically relevant because VDDR1A may initially resemble nutritional rickets, particularly in regions where vitamin D deficiency is common. However, the combination of hypocalcemia, hypophosphatemia, secondary hyperparathyroidism, elevated alkaline phosphatase, normal or near‐normal 25‐hydroxyvitamin D, and low or undetectable 1,25‐dihydroxyvitamin D should prompt consideration of a defect in vitamin D activation and early molecular confirmation when feasible [10, 11, 13]. Case 1 illustrates how diagnosis may be triggered by hypocalcemic seizures before severe deformity develops, whereas Case 2 illustrates the consequences of delayed recognition, with chronic progressive skeletal disease documented radiographically across multiple sites. Together, these cases emphasize the importance of considering VDDR1A in infants and children who present with seizures, hypotonia, delayed motor milestones, failure to thrive, or early skeletal deformities accompanied by biochemical evidence of rickets.

The radiographic findings in our patients further underscore the impact of disease duration and treatment continuity. Case 1 showed classic wrist rachitic changes at presentation, including widening of the distal radial and ulnar physes with metaphyseal cupping and fraying, but was diagnosed before major irreversible deformity had developed. In contrast, serial radiographs from Case 2 demonstrated persistent and multisite skeletal disease, including wrist involvement, forearm bowing, lower‐limb deformity, osteopenia, and thoracolumbar scoliosis. This radiographic progression provides a visual correlate of the prolonged metabolic insult associated with delayed diagnosis and poor long‐term adherence.

From a management perspective, these cases highlight the need not only for correct diagnosis but also for sustained long‐term follow‐up, family education, and reinforcement of adherence. Treatment with active vitamin D analogs such as calcitriol or alphacalcidol, together with adequate calcium supplementation, is generally effective in correcting biochemical abnormalities and improving skeletal mineralization [15]. However, the response depends on continuity of treatment and monitoring for biochemical normalization, growth, and evolving deformity [15]. In populations with a high prevalence of consanguinity, early recognition of inherited rickets, access to genetic testing, and counseling of affected families may be especially important for reducing diagnostic delay and preventing avoidable skeletal morbidity [16].

Our report has limitations. As a two‐patient case report, it cannot establish causal determinants of phenotype, and we were unable to directly investigate putative genetic or environmental modifiers. In addition, complete longitudinal radiographic documentation was not available for both cases to the same extent. Nevertheless, the side‐by‐side comparison of two children carrying the same homozygous CYP27B1 variant provides a clinically informative illustration of how markedly outcomes can diverge in VDDR1A and reinforces the importance of early diagnosis and consistent treatment.

## 4. Conclusion

These two cases highlight the marked clinical and radiographic variability of VDDR1A, even in children harboring the same homozygous CYP27B1 variant. Although genotype is central to disease pathogenesis, long‐term outcome appears to be shaped by a combination of diagnostic timing, treatment adherence, and likely additional biological and environmental modifiers. Early recognition of VDDR1A, prompt initiation of alphacalcidol with calcium supplementation, and sustained long‐term follow‐up can prevent progression to severe skeletal deformity and growth failure. In contrast, delayed diagnosis and inconsistent therapy may result in irreversible musculoskeletal complications. These observations support maintaining a high index of suspicion for VDDR1A in children presenting with hypocalcemia, seizures, developmental delay, or early rachitic changes, particularly in populations with frequent consanguinity.

## Funding

No funding was received for this manuscript.

## Ethics Statement

Ethical approval is not required for this study in accordance with local or national guidelines.

## Consent

Written informed consent was obtained from the patient for publication of the details of their medical case and any accompanying images.

## Conflicts of Interest

The author declares no conflicts of interest.

## Data Availability

The data that support the findings of this study are available from the corresponding author upon reasonable request.
